# Achilles tendon shear wave speed tracks the dynamic modulation of standing balance

**DOI:** 10.14814/phy2.14298

**Published:** 2019-12-09

**Authors:** Samuel A. Acuña, Anahid Ebrahimi, Robin L. Pomeroy, Jack A. Martin, Darryl G. Thelen

**Affiliations:** ^1^ Department of Physical Medicine & Rehabilitation University of Texas Southwestern Medical Center Dallas TX USA; ^2^ Department of Mechanical Engineering University of Wisconsin–Madison Madison WI USA; ^3^ Department of Materials Science and Engineering University of Wisconsin–Madison Madison WI USA; ^4^ Department of Orthopedics and Rehabilitation University of Wisconsin–Madison Madison WI USA; ^5^ Department of Biomedical Engineering University of Wisconsin–Madison Madison WI USA

**Keywords:** Achilles tendon force, center of pressure, posturography, shear wave tensiometer

## Abstract

Standing balance performance is often characterized by sway, as measured via fluctuations of the center of pressure (COP) under the feet. For example, COP metrics can effectively delineate changes in balance under altered sensory conditions. However, COP is a global metric of whole‐body dynamics and thus does not necessarily lend insight into the underlying musculotendon control. We have previously shown that shear wave tensiometers can track wave speeds in tendon as a surrogate measure of the load transmitted by the muscle‐tendon unit. The purpose of this study was to investigate whether shear wave metrics have sufficient sensitivity to track subtle variations in Achilles tendon loading that correspond with postural sway. Sixteen healthy young adults (26 ± 5 years) stood for 10 s with their eyes open and closed. We simultaneously recorded COP under the feet and shear wave speed in the right Achilles tendon. We found that Achilles tendon shear wave speed closely tracked (*r* > 0.95) dynamic fluctuations of the COP in the anteroposterior direction. Achilles tendon wave speed fluctuations significantly increased during standing with eyes closed, mirroring increases in COP fluctuations. These results demonstrate that tendon wave speed can track the subtle variations in Achilles tendon loading that modulate COP in standing. Hence, shear wave tensiometry exhibits the sensitivity to investigate the muscular control of quiet standing, and may also be useful for investigating other fine motor and force steadiness tasks.

## Graphical abstract

We show that Achilles tendon shear wave speed can track the subtle variations in tendon loading that modulate sway during quiet standing. Tendon wave speed exhibited sufficient sensitivity to track increases in tendon loading when standing with reduced sensory input. Hence, shear wave tensioimetry represents a new tool for investigating postural control and the muscle actions underlying other fine motor and force steadiness tasks.

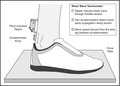

## 1 INTRODUCTION

The body undergoes continuous motion or sway when standing upright. Consequently, standing balance can be assessed by tracking fluctuations in the center of pressure (COP) under the feet (Duarte & Freitas, 2010; Duarte, Freitas, & Zatsiorsky, 2011; Hufschmidt, Dichgans, Mauritz, & Hufschmidt, 1980). Summary metrics of COP can effectively delineate changes in postural control arising from altered sensory conditions (Nagano, Yoshioka, Hay, & Fukashiro, 2006; Ruhe, Fejer, & Walker, 2010) and due to pathological conditions (Boisgontier et al., 2013; Hufschmidt et al., 1980; Salavati et al., 2009; Simoneau, Ulbrecht, Derr, Becker, & Cavanagh, 1994). However, the COP is inherently a measure of whole‐body dynamics and thus provides limited information about the underlying control at the muscular level. Characterizing the kinematic excursions that muscles undergo during standing (Loram, Maganaris, & Lakie, 2004, 2005, 2005) has provided insights into the sensory cues that are being used to modulate muscle‐tendon actions. However, to date, it has remained challenging to simultaneously monitor the forces transmitted by muscles.

We have recently shown that tendon shear wave speed can be measured and used as a proxy of muscle–tendon loading (Martin et al., 2018). Tendon wave speeds are measured using a shear wave tensiometer, which consists of a micron‐scale tapping device and two in‐series accelerometers secured on the surface of the skin overlying the tendon. Prior studies have shown a strong relationship between Achilles tendon wave speeds and net ankle kinetics during locomotion (Keuler, Loegering, Martin, Roth, & Thelen, 2019; Martin et al., 2018). However, it is not yet known whether wave speed exhibits the sensitivity needed to track subtle fluctuations in muscle‐tendon loading, which has relevance for studying postural control, force steadiness, and fine motor control tasks. Thus, the purpose of this study was to investigate whether Achilles tendon wave speed can track fluctuations in muscle loading during quiet standing in healthy young adults. We hypothesized that: (a) wave speed would closely track dynamic fluctuations of the COP that arise with modulation of Achilles tendon force, and (b) summary metrics of wave speed fluctuations would capture changes in postural sway that occur in the absence of vision.

## 2 METHODS

### 2.1 Participants

Sixteen healthy young adults participated in the study (8 female, mean ± *SD* age: 26 ± 5 years, height: 1.76 ± 0.11 m, mass: 74.2 ± 14.2 kg). Subjects were included in the study if they reported no current orthopedic or neurological impairments and no history of Achilles tendinopathy. The experimental protocol was approved by the University of Wisconsin–Madison Health Sciences Institutional Review Board, and all subjects provided written informed consent before participating in the study.

### 2.2 Experimental protocol

Subjects stood on a pair of force plates (BP400600‐2000, AMTI, Watertown, MA) for 10 s with their eyes open and closed. Subjects performed at least two trials of each condition, presented in random order. All subjects wore comfortable athletic shoes for the duration of the protocol, and stood with each foot on an individual force plate. We simultaneously recorded both the ground reaction force under each foot (1900 Hz) and the shear wave speed in the right Achilles tendon.

### 2.3 COP measurement and analysis

We recorded COP trajectories and ground reaction forces under the right foot, and additionally calculated net COP trajectories and net ground reaction forces using data from both force plates together (Winter, Prince, Stergiou, & Powell, 1993). We then determined the mediolateral and anteroposterior components of the COP (COP_ML_, COP_AP_) and vertical ground reaction force (F_z_). We low‐pass filtered (10 Hz) all COP data using a fourth‐order, zero‐lag Butterworth filter (Ruhe et al., 2010). We then centered the mean COP at zero in the ML and AP directions (Duarte & Freitas, 2010). We summarized the fluctuations of the COP using four conventional metrics: (a) the mean radial displacement of the COP from center, (b) the standard deviation of COP_AP_, (c) the mean velocity of the COP_AP_ trajectory, and (d) the range of the COP_AP_ (Baig, Dansereau, Chan, Remaud, & Bilodeau, 2012; Duarte et al., 2011; Raymakers, Samson, & Verhaar, 2005; Schubert, Kirchner, Schmidtbleicher, & Haas, 2012).

### 2.4 Shear wave tensiometry

**Figure 1:**
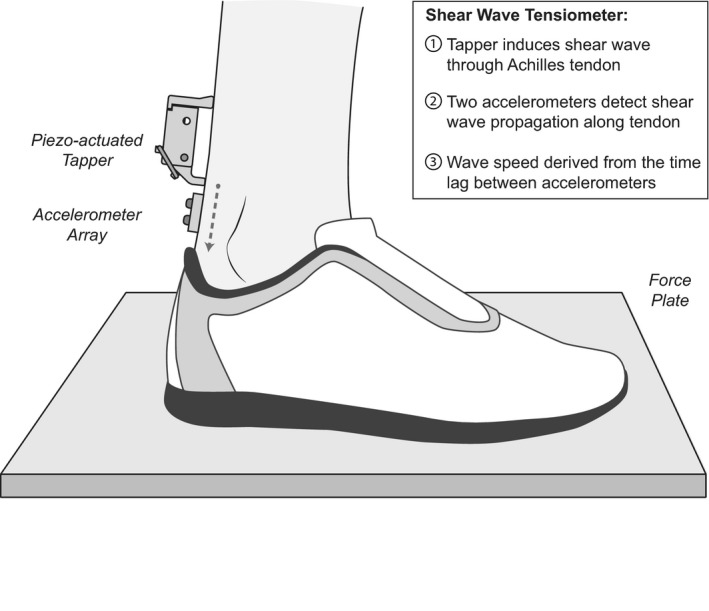
We simultaneously collected Achilles tendon shear wave speed and center of pressure under each foot when standing under eyes open and eyes closed conditions

We positioned a shear wave tensiometer (Martin et al., 2018) over each subject's right Achilles tendon (Figure 1). The tensiometer was secured over the tendon using double‐sided tape and self‐adhering wrap. The tensiometer consisted of a piezo‐actuated (PK4JQP2, Thorlabs, Newton, NJ) tapper and two miniature accelerometers (Model 352C23, PCB Piezotronics, Depew, NY) placed in series with the tapper and spaced 10 mm apart. The tapper was mounted in a 3D‐printed housing and driven by a 50 Hz square wave amplified via an open‐loop piezo controller (MDT694B, Thorlabs, Newton, NJ). The tapper delivered 50 micron impulsive taps. The resulting transient waves were recorded via the near and far accelerometers at 50,000 Hz. We then band‐pass filtered the accelerometer data using a second‐order, zero‐lag Butterworth filter. The lower cut‐off frequency was set to 150 Hz. The higher cutoff frequency was set to 5,000 Hz (12 subjects) or 1,500 Hz (4 subjects) to compensate for differences in the signal‐to‐noise ratio between subjects, which potentially arose due to differences in natural damping by the soft tissue overlying the tendon. All cutoff frequencies were consistently maintained between trials for individual subjects.

Shear wave speed was computed by ascertaining the time taken for the wave to traverse the distance between accelerometers. This was done by finding the time delay that maximized the normalized cross‐correlation between the two accelerometer signals over the first 1–3 ms after each tap event. Sub‐sample estimation of the time delay was found using a local 3‐point cosine fit of the normalized cross‐correlation values (Céspedes, Huang, Ophir, & Spra 1995). We computed Achilles tendon shear wave speed by dividing the inter‐accelerometer distance (10 mm) by this wave travel time. We then low‐pass filtered (10 Hz) the wave speed signals using a fourth‐order, zero‐lag Butterworth filter. We quantified fluctuations in Achilles tendon shear wave speed using summary metrics analogous to our COP summary metrics: (a) the mean absolute deviation, (b) the standard deviation, (c) the mean rate of change (i.e. the time derivative of wave speed), and (d) the range of wave speed. We normalized the summary metrics to the average wave speed of each trial.

We used spectral analysis to compare the similarity of the COP and wave speed signals at frequencies ranging up to 10 Hz. To do this, we computed the energy‐normalized power spectral density of the zero‐order detrended COP and wave speed data during the eyes open condition. We also used the wave speed data to estimate the absolute tendon forces during standing. To do this, we used the average tensiometer calibration parameters and Achilles tendon geometries from a prior study of 12 healthy young adults performing isometric ankle plantarflexion on a dynamometer (Keuler et al., 2019). We then conducted a fast Fourier transform (FFT) of the detrended tendon force to ascertain the magnitude of force fluctuations measured at frequencies ranging up to 10 Hz.

### 2.5 Statistical analysis

To address our first hypothesis, Pearson's correlations examined the relationship between Achilles tendon shear wave speed and COP_AP_ for every trial. We also compared the normalized power spectral density of Achilles tendon wave speed and COP_AP_ using a Wilcoxon signed‐rank test, computed over five frequency bands ranging from 0–10 Hz. We similarly compared the magnitudes of tendon force between the eyes open and closed conditions over these frequency bands. To address our second hypothesis, a series of paired‐samples *t‐*tests assessed differences between visual conditions (eyes open, closed) in our summary metrics of COP and Achilles tendon wave speed. For these comparisons, we controlled the false discovery rate using the Benjamini–Hochberg procedure (Benjamini & Hochberg, 1995). Shapiro–Wilk tests confirmed assumptions of normality for all statistical tests. Measures for repeated trials were averaged together for each subject. We performed all the statistical analyses using SPSS (v.25, IBM Corp., Armonk, NY), and defined significance a priori as *p* < .05.

## 3 RESULTS

**Figure 2:**
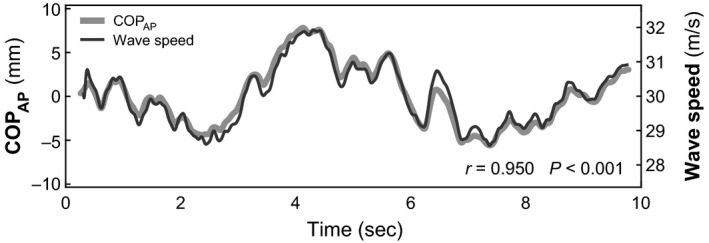
Achilles tendon shear wave speed was highly correlated with fluctuations in the sagittal center of pressure under one foot, as shown in this representative trial

We examined relationships within 64 trials of quiet standing. Shear wave speed for the right Achilles tendon was highly correlated with the COP_AP_ under the right foot (mean *r* = .96, range = 0.86–0.99, *p* < .001) (Figure 2). Shear wave speed was also significantly correlated with the net COP_AP_ (mean *r* = .94, range = 0.83 to 0.99, *p* < .001).

**Figure 3:**
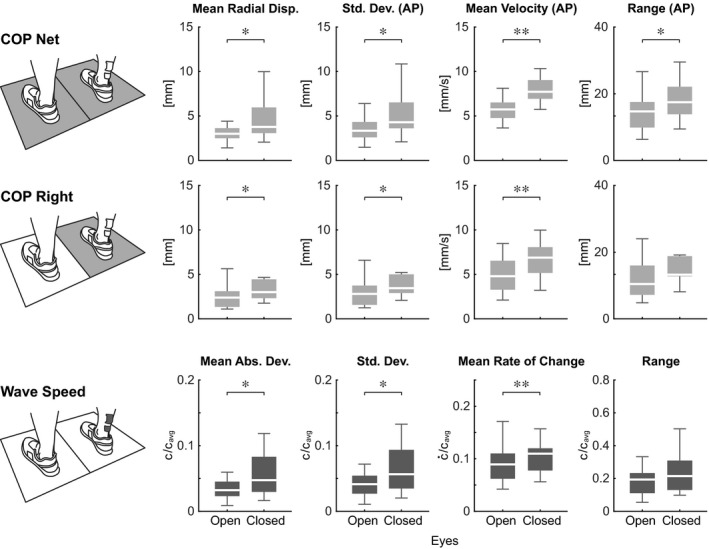
Fluctuations of Achilles tendon shear wave speed (c) and center of pressure (COP) increased when subjects stood with their eyes closed, compared to eyes open. Boxplots represent variation of the summary metrics across subjects. Asterisks indicate significant differences between conditions, found using paired‐sample *t*‐tests with 16 subjects, where *= *p* < .05 and **= *p* < .01

Summary metrics of COP and Achilles tendon shear wave speed increased when subjects stood with their eyes closed compared to eyes open (Figure 3). We observed a significant increase from the eyes open condition in the mean radial displacement of the net COP (+37%, *p* = .008, $\eta_{p}^{2}$ = 0.43) as well as the COP under the right foot (+37%, *p* = .028, $\eta_{p}^{2}$ = 0.32). We also saw significant increases in the standard deviation of the COP_AP_ (net: +35%, *p* = .014, $\eta_{p}^{2}$ = 0.38; right: +34%, *p* = .037, $\eta_{p}^{2}$ = 0.29), as well as the mean velocity of the COP_AP_ (net: +39%, *p* < .001, $\eta_{p}^{2}$ = 0.84; right: +34%, *p* < .001, $\eta_{p}^{2}$ = 0.68). The range of the COP_AP_ also increased, however this was only significant for the net COP (net: +26%, *p* = .049, $\eta_{p}^{2}$ = 0.27; right: +23%, *p* = .097, $\eta_{p}^{2}$ = 0.20). Similarly, in our summary metrics of normalized Achilles tendon shear wave speed, the eyes closed condition significantly increased the mean absolute deviation (+67%, *p* = .024, $\eta_{p}^{2}$ = 0.33), the standard deviation (+55%, *p* = .033, $\eta_{p}^{2}$ = 0.31), and the mean rate of change (+23%, *p* = .009, $\eta_{p}^{2}$ = 0.42). The range of the normalized shear wave speed tended to increase in the eyes closed condition, but did not reach significance (+33%, *p* = .082, $\eta_{p}^{2}$ = 0.22).

**Figure 4:**
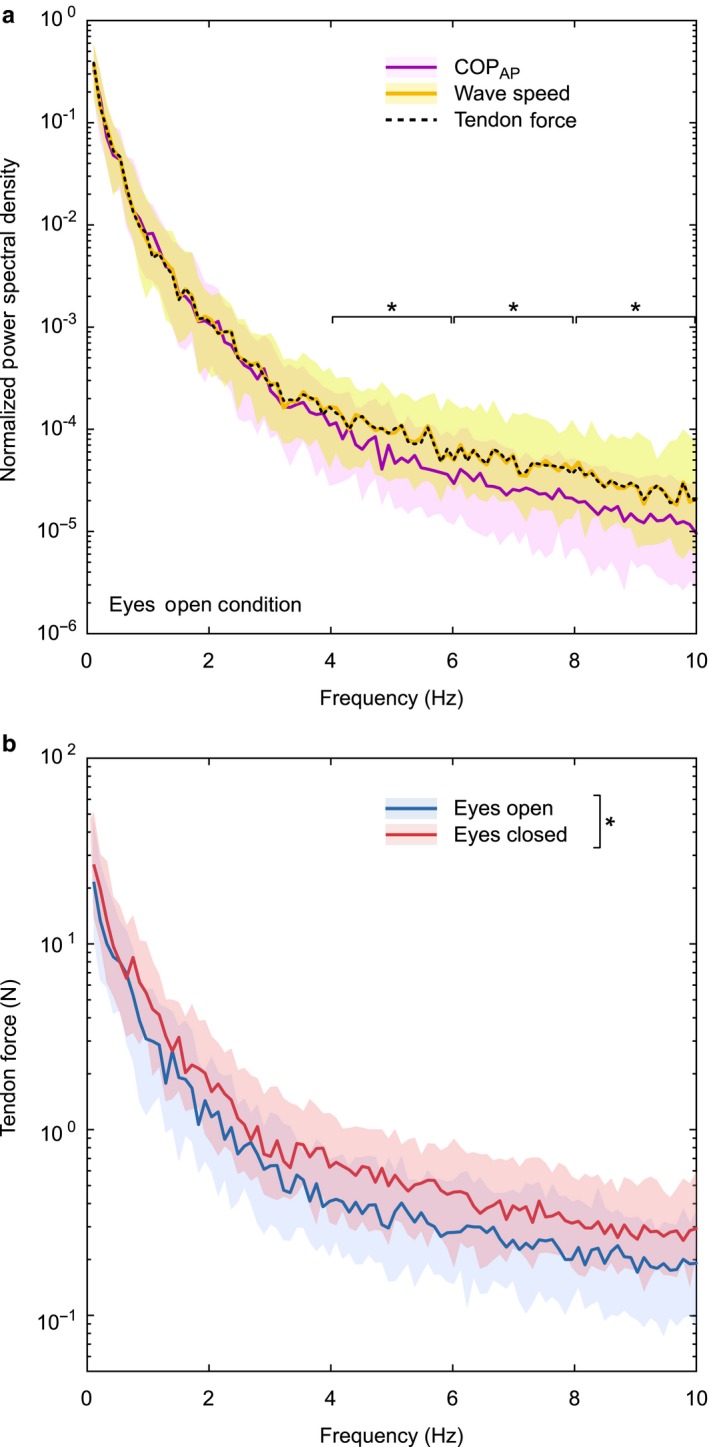
Spectral patterns of the center of pressure (COP), Achilles tendon wave speed, and Achilles tendon force. (a) Power spectral density normalized to the total energy of each signal during the eyes open condition. (b) Achilles tendon force was estimated from wave speed using average calibration parameters and tendon geometry from Keuler et al. (2019). The shaded curves represent the interquartile range of the spectrum and the solid and dashed lines represent the medians. Asterisks in (a) indicate significant differences between median wave speed and COP signal power over a 2 Hz frequency band, found using Wilcoxon signed‐rank tests. The asterisk in (b) indicates that we found significant differences in median tendon force between the eyes open and closed conditions for each 2 Hz frequency band

Spectral analysis revealed that Achilles tendon shear wave speed and COP exhibited similar spectral patterns in the 0–2 Hz band (*p* = .907) and 2–4 Hz band (*p* = .350). However, shear wave signals exhibited relatively more signal power than the COP within the 4–6, 6–8, and 8–10 Hz frequency bands (*p* < .001) (Figure 4a). Achilles tendon force fluctuations exhibited a median value of 7.7 *N* (interquartile range, IQR: 3.4–16.4 N) at frequencies < 1 Hz (Figure 4b). Smaller Achilles tendon force fluctuations of < 1 N were measured at higher (4–10 Hz) frequencies. For every frequency band, median Achilles tendon force was significantly greater during the eyes closed condition compared to the eyes open condition (*p* < .009) (Figure 4b).

## 4 DISCUSSION

We investigated the potential of using a shear wave tensiometer to track the muscular control of quiet standing. An inverted pendulum model is often used to describe quiet standing, with sway arising due to rotation about the ankle (Winter, 1995; Winter, Patla, Ishac, & Gage, 2003; Winter et al., 1993). Sway can be assessed by tracking fluctuations in the COP (Winter et al., 1993). Given that vertical force is relatively constant in quiet standing, the sagittal fluctuations in COP arise from variations in ankle torque. Hence for quiet standing with little antagonist co‐contraction, fluctuations in the sagittal COP represent modulation of Achilles tendon force via the triceps surae (Masani, Sayenko, & Vette, 2013; Winter, 1995; Winter et al., 1993). Thus, the strong correlation between wave speed and COP observed in this study is strong evidence of the capacity for using shear wave tensiometry to track subtle variations in muscle loading underlying standing balance control.

In support of our second hypothesis, Achilles tendon wave speed metrics captured sensory‐induced changes in standing performance. For example, the rate of change in normalized shear wave speed increased significantly when subjects closed their eyes while standing. Similarly, we observed increases in the velocity of the COP, which is considered one of the most informative summary metrics of standing balance performance (Baig et al., 2012; Raymakers et al., 2005). These results suggest that shear wave tensiometers may have sufficient sensitivity to delineate changes in standing balance due to altered sensory conditions that arise due to aging (Acuña, Francis, Franz, & Thelen, 2019; Hortobágyi et al., 2009; Peterson & Martin, 2010) or pathological conditions.

There were slight differences in the spectral characteristics of wave speed and COP (Figure 4a). In particular, tendon wave speed exhibited greater normalized signal power than the COP at the upper frequencies (i.e., 4–10 Hz) of the postural domain (Ruhe et al., 2010). This result suggests that higher frequency tendon forces may be naturally attenuated by the dynamics of the musculoskeletal system. The magnitude of tendon force fluctuations in quiet standing ranged from ~10 N at low frequencies (<1 Hz) to less than 1 N at frequencies above 4 Hz (Figure 4b). Tendon force fluctuations increased in the eyes closed condition, which may give rise to the enhanced COP velocity observed in that condition. Thus, shear wave tensiometry represents a viable approach for quantitatively assessing the small fluctuations in tendon loading that can arise in postural control, and may extend to other fine motor and force steadiness tasks.

The force sensitivity of the tensiometer technology brings about unique opportunities in biomechanics and motor control. For example, tensiometers could be used to directly assess subtle fluctuations in muscle–tendon loading during postural control, force steadiness experiments, and fatigue studies (Bandholm, Rose, SlØk, Sonne‐Holm, & Jensen, 2009; Shinohara, Yoshitake, Kouzaki, & Fukunaga, 2006; Tracy, 2007). The technology may be particularly important in pathological populations, where the relationship between external loading and internal muscle forces is challenging to deduce. Tendon force data can also be coupled with measures of internal muscle kinematics (Loram et al., 2004, 2005, 2005) to evaluate the sensory cues underlying fine motor control. Finally, tensiometers can be deployed on multiple tendons (e.g., patellar and hamstrings; Martin et al., 2018) to investigate the active control of multiple joints, which is relevant to more challenging postural control tasks involving altered external environments and support surfaces (Acuña, Zunker, & Thelen, 2019; Dickin & Doan, 2008).

This study has some limitations to consider. First, we derived our summary metrics of COP and wave speed using only ten seconds of collected data, which is a relatively short time window for posturography (Duarte & Freitas, 2010). While these collections were sufficient to demonstrate promise, extended collections are needed to more fully evaluate repeatability, amplitude, and frequency characteristics of tensiometer signals across a range of postural tasks. The Achilles tendon wave speed measure represents a measure of net load induced by the triceps surae muscles. Further work is needed to ascertain how one might decompose the individual muscle contributions from the net tendon force. Finally, the present study used shear wave speed as a proxy measure of tendon loading. While wave speed metrics were sufficient to distinguish standing performance between conditions (eyes open, eyes closed), subject‐specific calibration procedures should be performed to assess absolute tendon loading in an individual (Keuler et al., 2019). The relationship between stress and wave speed is considered quadratic (Martin et al., 2018), and we are justified if instead we choose to use wave speed squared as our indirect measure of tendon loading. For simplicity, our study used wave speed, but we found similar results when using wave speed squared (e.g., correlations between wave speed squared and COP_AP_ under the right foot: mean *r* = .96, range = 0.85–0.99).

One potential concern with using a shear wave tensiometer over the Achilles tendon is that the vibration required to generate shear waves may in fact alter postural control. With a sufficiently high intensity, Achilles tendon vibration is known to induce a whole‐body posterior tilt during quiet standing (Adamcova & Hlavacka, 2007; Barbieri, Gissot, Nougier, & Pérennou, 2013; Capicikova, Rocchi, Hlavačka, Chiari, & Cappello, 2006; McKay, Wu, & Angulo‐Barroso, 2014). To examine whether the shear wave tensiometer might alter COP trajectories during quiet standing, we conducted a small, exploratory study using eight individuals, four of which participated in the primary study (see Data S1). We found that the active tensiometer did not significantly shift the mean COP_AP_, and that the summary metrics of COP appeared to be unaffected by the tensiometer. This is likely because the amplitude of the tensiometer taps is relatively small (<50 μm). Further study of this issue may be warranted when extending the technology to other populations.

In conclusion, our results provide evidence that subtle fluctuations in Achilles tendon loading during quiet standing can be tracked by measuring shear wave speed in the tendon. Additionally, wave speed fluctuations were sufficient for quantifying altered balance performance without visual feedback. Hence, shear wave tensiometry exhibits the sensitivity to investigate the muscular control of quiet standing, and may also be useful for investigating other fine motor and force steadiness tasks.

## CONFLICT OF INTEREST

Two of the authors (J.A.M., D.G.T.) are co‐inventors on a pending patent application for technology relating to the methods described herein. The other authors declare no conflict of interest.

## AUTHOR CONTRIBUTIONS

All authors were fully involved in the study and in the preparation of the manuscript.
