# Supplementary material for: Achilles tendon shear wave speed tracks the dynamic modulation of standing balance
Source: Physiol Rep. 2019 Dec 9;7(23):e14298. doi: 10.14814/phy2.14298 (PMC6900496; doi:10.14814/phy2.14298)
Supplement: Supplementary file 1 [file PHY2-7-e14298-s001.docx]

**Achilles Tendon Shear Wave Speed Tracks the Dynamic Modulation of Standing Balance
*Supplementary Info***

Samuel A. Acuña^1^, Anahid Ebrahimi^1^, Robin L. Pomeroy^1^, Jack A. Martin^3^, Darryl G. Thelen^1234^

^1^Department of Mechanical Engineering

^2^Department of Biomedical Engineering

^3^Department of Materials Science and Engineering

^4^Department of Orthopedics and Rehabilitation

University of Wisconsin–Madison, Madison, WI, USA

**Research Question**

Does the tapping device used in a shear wave tensiometer significantly alter standing balance, as measured via center of pressure (COP) trajectories during quiet standing?

Hypotheses:

1. The shear wave tensiometer will not affect the summary metrics of COP fluctuations.
2. Turning on the shear wave tensiometer during quiet standing will not cause the COP to shift in the anteroposterior direction (COP_AP_).
3. After using the shear wave tensiometer during quiet standing, turning off the tensiometer will not cause a shift in the COP_AP_.

**Methods**

Participants

Eight healthy young adults participated in this study (2 female, mean ± SD age: 24 ± 3 yrs, height: 1.86 ± 0.09 m, mass: 80.9 ± 8.4 kg), four of which were participants in the main study presented in the manuscript. Subjects were included in the study if they reported no current orthopedic or neurological impairments, reported no history of Achilles tendinopathy, and were not pregnant. The experimental protocol was approved by the University of Wisconsin–Madison Health Sciences Institutional Review Board, and all subjects provided written informed consent before participating in the study.

Experimental Protocol

We placed a shear wave tensiometer over the right Achilles tendon of each participant. Subjects then stood barefoot on a low-profile balance plate (Legacy Balance Plate, Bertec Corp., Columbus, OH) and completed two separate testing protocols. The first protocol examined whether the shear wave tensiometer affects the summary metrics of COP fluctuations. Subjects stood for 30 seconds with their eyes open and closed and with the shear wave tensiometer on and off. Subjects performed three trials for each condition for a total of 12 trials, presented in random order. The second protocol examined whether the shear wave tensiometer causes a shift in the COP. Again, subjects stood for 30 seconds their eyes open and closed and with the shear wave tensiometer on and off. However, in this case, if the tensiometer was to be on during a trial, it was only turned on for the middle 10 seconds of that trial (1, 3, 4, 7). Subjects performed three trials of each condition for a total of 12 trials, presented in random order. To counter the effects of fatigue in both these protocols, we required participants to step off the balance plate and rest between each trial.

Measurement and Analysis

We acquired (1000 Hz) the net COP from the balance plate during the two separate testing protocols. We low-pass filtered (10 Hz) the COP data using a fourth-order, zero-lag Butterworth filter. We then examined the COP data from the first protocol. Here, we centered the mean COP at zero in the ML and AP directions (5). We summarized the fluctuations of the COP using five conventional metrics: 1) the mean radial displacement of the COP from center, 2) the standard deviation of COP_AP_, 3) the mean velocity of the COP_AP_ trajectory, 4) the range of the COP_AP_ , and 5) the area covered by the COP displacement (defined as an ellipse containing 95% of the COP trajectory) (2, 6, 8, 9). Next, we examined the COP_AP_ data from the second protocol. Here, we averaged the COP_AP_ trajectories within three time intervals: 5–10 sec, 15–20 sec, and 25–30 sec. We then computed the difference in mean COP_AP_ between intervals 1 and 2 (∆COP_2_), and between intervals 2 and 3 (∆COP_3_).

Statistical Analysis

First, to assess whether the shear wave tensiometer affects the summary metrics of COP fluctuations (Hypothesis 1), a two-way repeated-measures analysis of variance tested for main effects of and interactions between the shear wave tensiometer (turned on, off) and visual conditions (eyes open, closed) on our summary metrics of the net COP fluctuations. Second, to assess whether turning on the shear wave tensiometer shifted the COP_AP_ (Hypothesis 2), a paired-samples *t-*test assessed the difference in ∆COP_2_ between when the tensiometer was turned on or remained off (i.e. the control condition). Third, to assess whether turning off the shear wave tensiometer shifts the COP_AP_ (Hypothesis 3), a paired-samples *t-*test assessed the difference in ∆COP_3_ between turning the tensiometer off and the control condition. Shapiro-Wilk tests confirmed assumptions of normality for all statistical tests. Measures for repeated trials were averaged together for each subject. We performed all the statistical analyses using SPSS (v.25, IBM Corp., Armonk, NY), and defined significance *a priori* as *p* < 0.05.

**Results**

We observed significant main effects of visual condition (eyes open, closed) on all the summary metrics of net COP fluctuations (Table 1). We found no interaction effects between the tensiometer and visual conditions. Only one summary metric, the mean radial displacement, revealed a small (< 0.5 mm) yet significant main effect related to the shear wave tensiometer (*p* = 0.026).

Turning on or turning off the shear wave tensiometer during quiet standing elicited no significant shifts in the mean COP_AP_, compared to the control condition (Table 2).

**Discussion**

In support of our first hypothesis, the shear wave tensiometer did not significantly affect most of our summary metrics of COP fluctuations. Only one summary metric showed a significant main effect due to the tensiometer: the mean radial displacement (*p* = 0.026). However, considering the average difference in mean radial displacement was very small (< 0.5 mm), we interpret this summary metric of COP to be mostly unaffected by the tensiometer.

In support of our second and third hypotheses, turning the shear wave tensiometer on or off did not elicit a significant shift in the mean COP_AP_. However, our data suggests that turning off the tensiometer might cause a small shift in the mean COP_AP_, as the difference between active and control during the eyes closed condition was trending towards significance (average difference: 2.6 ± 1.6 mm, *p* = 0.073), although both active and control shifted in the same direction. Regardless, we interpret our results to suggest that if the tensiometer does indeed cause a shift in the COP, it is certainly very small, and no more than a few millimeters.

Achilles tendon vibration is well-known to induce a whole-body backwards tilt during quiet standing (1, 3, 4, 7). This effect is usually achieved through applied vibration over the Achilles tendon with a relatively high intensity. As a result, turning on the vibration causes an obvious increasing posterior shift in the COP_AP_ (e.g. > 40 mm (3)), and turning off the vibration results in an overcorrection that shifts the COP_AP_ to be anterior to the pre-vibration COP_AP_ (e.g. 5-10 mm). We did not see evidence of any significant shifts in the COP_AP_ as a result of turning the vibration on or off. Our results suggest that the resulting Achilles tendon vibration when using the shear wave tensiometer does not induce a posterior whole-body tilt, likely because the intensity of vibration here is very small relative to the intensity that induces a shift.

There are limitations with this exploratory study. We only used one shear wave tensiometer over the right Achilles tendon, and we recognize that the destabilizing effect of Achilles tendon vibration could be stronger with bilateral vibration. Further, we only considered the net COP under both feet, which may not accurately reflect changes in COP under the foot on which we applied the tensiometer.

We conclude that the micron scale taps applied by a shear wave tensiometer do not significantly alter quiet standing balance in healthy young adults.

Table 1. ﻿Group mean (± standard deviation) values for the summary metrics of center of pressure (COP). We also report main effects and interactions between tensiometer and visual conditions using a repeated measures analysis of variance. N = 8.

|  |  | Vision | |  |  |  |  |  |
| --- | --- | --- | --- | --- | --- | --- | --- | --- |
| Summary Metric of COP | Tensiometer | Eyes Open | Eyes Closed |  | Effect | *F-*Statistic | *p-*Value | Partial η^2^ |
| Mean Radial Displacement [mm] | Off | 2.8 (0.7) | 3.7 (1.1) |  | Tensiometer | 7.884 | **0.026** | 0.530 |
|  | On | 2.5 (0.6) | 3.3 (1.0) |  | Vision | 16.593 | **0.005** | 0.703 |
|  |  |  |  |  | Tensiometer*Vision | 0.048 | 0.833 | 0.007 |
| Standard Deviation (AP) [mm] | Off | 2.9 (0.7) | 4.0 (1.2) |  | Tensiometer | 3.043 | 0.125 | 0.303 |
|  | On | 2.7 (0.6) | 3.8 (1.2) |  | Vision | 14.399 | **0.007** | 0.673 |
|  |  |  |  |  | Tensiometer*Vision | 0.016 | 0.904 | 0.002 |
| Mean Velocity (AP) [mm/s] | Off | 6.4 (1.1) | 9.5 (2.5) |  | Tensiometer | 0.784 | 0.405 | 0.101 |
|  | On | 6.2 (1.6) | 9.3 (2.6) |  | Vision | 37.484 | **<0.001** | 0.843 |
|  |  |  |  |  | Tensiometer*Vision | 0.019 | 0.895 | 0.003 |
| Range (AP) [mm] | Off | 15.7 (3.8) | 21.9 (7.1) |  | Tensiometer | 0.027 | 0.873 | 0.004 |
|  | On | 14.7 (4.3) | 22.4 (8.7) |  | Vision | 15.647 | **0.005** | 0.691 |
|  |  |  |  |  | Tensiometer*Vision | 0.260 | 0.626 | 0.036 |
| Area [mm^2^] | Off | 81.5 (40.7) | 128.5 (92.5) |  | Tensiometer | 1.960 | 0.204 | 0.219 |
|  | On | 61.5 (38.0) | 117.9 (97.3) |  | Vision | 5.958 | **0.045** | 0.460 |
|  |  |  |  |  | Tensiometer*Vision | 0.803 | 0.400 | 0.103 |

Table 2. Group mean (± standard deviation) values for the change in mean center of pressure in the anteroposterior direction after turning the shear wave tensiometer on (∆COP_2_) and off (∆COP_3_). Significance in difference between active and control conditions reported as *p-*values from paired-samples *t*-tests. N = 8.

|  | ∆COP_2_ [mm] | |  |  | ∆COP_3_ [mm] | |  |
| --- | --- | --- | --- | --- | --- | --- | --- |
| Vision | Active | Control | *p*-Value |  | Active | Control | *p*-Value |
| Eyes Open | -0.6 (3.1) | -1.6 (3.3) | 0.327 |  | -0.6 (3.1) | -0.5 (2.7) | 0.879 |
| Eyes Closed | -2.4 (2.4) | -0.5 (3.0) | 0.200 |  | -3.4 (2.3) | -0.6 (3.9) | 0.073 |

**References**

1. **Adamcova N**, **Hlavacka F**. Modification of human postural responses to soleus muscle vibration by rotation of visual scene. *Gait Posture* 25: 99–105, 2007.

2. **Baig S**, **Dansereau RM**, **Chan ADC**, **Remaud A**, **Bilodeau M**. Cluster Analysis of Center-of-Pressure Measures. *Int J Electr Comput Eng* 1, 2012.

3. **Barbieri G**, **Gissot A-S**, **Nougier V**, **Pérennou D**. Achilles tendon vibration shifts the center of pressure backward in standing and forward in sitting in young subjects. *Neurophysiol Clin Neurophysiol* 43: 237–242, 2013.

4. **Capicikova N**, **Rocchi L**, **Hlavačka F**, **Chiari L**, **Cappello A**. Human postural response to lower leg muscle vibration of different duration. *Physiol Res* 55: 1–6, 2006.

5. **Duarte M**, **Freitas SMSF**. Revision of posturography based on force plate for balance evaluation. *Rev Bras Fisioter* 14: 183–192, 2010.

6. **Duarte M**, **Freitas SMSF**, **Zatsiorsky V**. Control of Equilibrium in Humans - Sway over sway. In: *Motor Control: Theories, Experiments, and Applications*, edited by Danion F, Latash ML. New York, NY: Oxford University Press, 2011, p. 219–242.

7. **McKay SM**, **Wu J**, **Angulo-Barroso RM**. Effect of Achilles tendon vibration on posture in children. *Gait Posture* 40: 32–37, 2014.

8. **Raymakers J a.**, **Samson MM**, **Verhaar HJJ**. The assessment of body sway and the choice of the stability parameter(s). *Gait Posture* 21: 48–58, 2005.

9. **Schubert P**, **Kirchner M**, **Schmidtbleicher D**, **Haas CT**. About the structure of posturography: Sampling duration, parametrization, focus of attention (part I). *J Biomed Sci Eng* 05: 496–507, 2012.
